# Risk factors and complications of puerperal sepsis at a tertiary healthcare centre

**DOI:** 10.12669/pjms.294.3389

**Published:** 2013

**Authors:** Meharun-Nissa Khaskheli, Shahla Baloch, Aneela Sheeba

**Affiliations:** 1 Dr. Meharun-Nissa Khaskheli, MBBS, FCPS, Associate Professor, Department of Obstetrics & Gynaecology, Liquat University of Medical & Health Sciences, Jamshoro/Hyderabad, Sindh, Pakistan.; 2 Dr. Shahla Baloch, MBBS, DCH, FCPS, Associate Professor, Department of Obstetrics & Gynaecology, Liquat University of Medical & Health Sciences, Jamshoro/Hyderabad, Sindh, Pakistan.; 3 Dr. Aneela Sheeba, MBBS, DMRD, FCPS, Assistant Professor, Department of Obstetrics & Gynaecology, Liquat University of Medical & Health Sciences, Jamshoro/Hyderabad, Sindh, Pakistan.

**Keywords:** Puerperal sepsis, Risk factors, Complications

## Abstract

***Objective:*** To determine the risk factors and complications of puerperal sepsis.

***Methods:*** This was an observational prospective Cohort study conducted from January 2011 to December 2011 at the Obstetrics and Gynaecology Department Liaquat University of Medical & Health Sciences Jamshoro/Hyderabad, Sindh Pakistan. During this study period, all the women who delivered in this hospital or referred to this hospital within 42 days after delivery with puerperal pyrexia/sepsis diagnosed on clinical examination as well as with relevant investigations were included in the study. Women with other ailments like malaria, typhoid fever and postpartum eclampsia during the puerperal period were excluded. The subjects were registered on predesigned proforma after giving informed written consent. The data was collected and analyzed using SPSS version 17.

***Results: ***During this period there were 3316 obstetrical admission and out of these 129(3.89%) women had puerperal sepsis. Most of these women 84(65.11%) were aged 31 years and above, multiparous 101 (78.29%), and unbooked 98 (75.96%) cases. Common risk factors found were absent membranes in 108(83.72%) of the women, delivered or undelivered and mismanaged, referred cases 95(73.64%), are being delivered in this hospital 34(26.35%). Morbidities seen were septicemia in 35 (27.13%) cases, and disseminated intra vascular coagulation in 23(17.82%) cases, while 11 (8.52%) of the women died.

***Conclusion: ***Common risk factors were anaemia; suboptimal personal hygiene as well as improper sterilization which resulted in severe health hazards such as septicemia, disseminated intravascular coagulation as well as death.

## INTRODUCTION

According to The World Health Organization (WHO), puerperal sepsis is defined as the infection of the genital tract occurring at labour or within 42 days of the postpartum period. The puerperal sepsis/pyrexia presents commonly with fever and other symptoms like pelvic pain, foul smelling vaginal discharge and delayed reduction of the uterine size.^1^ World literature search revealed a Nigerian study^2^ report that puerperal sepsis is a second leading cause of death accounting for 26.3% of maternal deaths, while another WHO report estimated 358,000 maternal deaths yearly occurring due to child birth problems and out of these up to 15% are associated with puerperal sepsis.^3^

Dushyant D et al^4^ study reported that puerperal pyrexia and sepsis are highly preventable problems occurring among the leading causes of maternal morbidity and mortality not only in the developing countries but also in developed countries as well. Common predisposing factors leading to puerperal sepsis are anaemia, prolonged labour, frequent vaginal examinations in labour under unsterilized circumstances, premature rupture of membranes for prolonged period.^5^ Puerperal sepsis results from infection contacted during child birth and this is one of the commonest causes of maternal mortality in the developing countries. Despite the discovery of antibiotics over eighty years ago, there is still a strong need for their proper and prophylactic utilization. Some developing countries have experienced increased use of health facilities for labour and delivery care but there is a lack of proper monitoring or checks and balances and there is a possibility that this trend could lead to rising rates of puerperal sepsis. Drug and technological developments needs to be combined with effective health system intervention to reduce infection including puerperal sepsis.^6^

This highly important maternal issue requires special attention. There should be no excuse for delaying targeted, global action to implement and evaluate infection control measures during labour and delivery for the prevention and reduction of puerperal sepsis and other related conditions. The choice of the specific combination of components to be evaluated could be informed by what is known from the wider infection control literature, from exciting information on ways to improve quality in maternity care and by tailoring strategies to address underlying problems of infection control.^7^

Considering important maternal health issues, more recent studies have been conducted to establish the optimal knowledge and skills of infection prevention specialists and of staff to bed ratios but clear recommendations on effective organization of staff have not yet emerged.^8^ Some guidelines such as those on hand hygiene are highly specific and have been developed using quality assessed evidence meticulously gathered from reviews of literature. These have been the product of work done as part of the global patient safety challenge which targeted hand hygiene as a flagship campaign.^9^^,^^10^

The rationale of the study is analyses of various risk factors responsible for this hazardous maternal health issue with its related complications so as to take necessary action for its prevention.

## METHODS

Out of the total obstetrical admissions, 129 women were recruited for the study. The sample size was calculated empirically (The prevalence of the condition is 9.3% [By Pakistan demographic and health survey 2007:113, http://www.ayubmed.edu.pk/JAMC/Past/22-3/Shamshad] Confident interval 95%, Formula N= (Z)^2^(pq)/e^2^), the sampling technique is simple random. The selected population was admitted as emergencies in the labour ward, maternity ward, or through the outpatient department. They were evaluated thoroughly by the taking of detailed history and clinical examination. These women were having puerperal pyrexia/sepsis and delivered within 42 days, they were diagnosed on clinical examination and with relevant investigations like elevated body temperatures, abdominal distention,dehydration,foul smelling lochia,complete blood picture, rise in total leukocyte count, platelet count, co agulation profile, serum electrolytes, ultrasound examination for intra peritoneal collection,uterine collection, retained products of conception and those women with other problems like constipation, backache, chest.

Infection, malaria and typhoid fever were excluded from the study. The data of recruited subjects was registered on the predesigned proforma after taking informed written consent. The variables studied were demographic characteristics like age groups between 15 to 40 years, parity, primiparous as well as grandmultiparous women, booking status booked or unbooked, labour characteristics like onset of labour, status of membranes, mode of labour, place of delivery, investigations, clinical spectrum and maternal morbidity and mortality. The data was collected and analyzed on SPSS version 17. Statistical analyses for qualitative type of data were done by Chi square test. P value less than 0.05 was considered as highly significant. The result is presented in terms of simple percentage, relative risk, Chi square test P value.

## RESULT

Majority of the women admitted with puerperal sepsis were above thirty years of age 84(65.11%), other vulnerable group was below 20 years of age in 28 (21.70%) women, while between 21 years to 30 years of age group were 17 (13.17%) women (P value 0.5). Highly affected women were grand multiparous having parity 5 and above in 101 (78.29%) women, primiparous women were 20 (15.50%), (P value 0.5). Frequent problem was observed in unbooked population in 98 (75.96%) cases, booked women were 31 (24.03%), confidence interval 95% [Relative risk 3.1612], spontaneous onset of labour was observed in 77 (59.68%) women, labour was induced in 52 (41.31%) cases, confidence interval 95% [Relative risk 1.4807] (P value 0.05), 108 (83.72%) women were seen with absent membranes at the time of admission, they were having the history of leaking of liquor for 10 to 16 hours and on clinical examination membranes were absent, confidence interval 95%, confidence interval 95% [Relative risk 0.1944], p value 0.001, spontaneous vaginal delivery was observed in 78(60.46%) women, 22 (17.05%) women needed instrumental intervention due to prolonged second stage and 29 (22.48%) women had caesarean section due to cephalopelvic disproportion, fetal distress, obstructed labour, delivered or undelivered referred cases from periphery were 95 (73.64%) (Table-I).

Most of these women were anemic with haemoglobin level between 8.5 to 9 grams % were in 74 (57.36%) women, while 26 (20.15%) women were having haemoglobin level up to 5 grams % (P value 0.5), total leukocyte count was more than 11000/cumm^2^ in 93 (72.09%) cases, high vaginal swab culture and sensitivity report was seen positive in 72 (55.81%) cases, while in 57(44.18%) women no bacterial growth was reported, confidence interval 95% [Relative risk 1.2631] (P value 0.05) (Table-II).

Common presenting symptoms were fever in 117 (90.69%) women, wound infection in 51 (39.53%) women, and abdominal distention in 28 (21.70%) women, majority of these women were hospitalized for more than 10 days 109 (85.82%). Common morbidities seen were disseminated intravascular coagulation in 23 (17.82%) women, septicemia in 35 (27.13%) women, while 11 (8.52%) women died (Table-III).

**Table-I:** Socio demographic characteristics (N=129).

*S. No*	*Socio demographic Characteristics*	*No of Cases (%)*	*Relative Risk*	*Chi Squire Test*	*P value*
1	Age:				
	a. Below 20 years	28(21.70%)	-	0.0798	0.5
	b. Between 21-30 years	17(13.17%)	-		
	c. 31 and above years	84(65.11%)	-		
2	Parity:				
	a. Primiparous	20(15.50%)	-	0.0798	0.5
	b. Para 1-4	8(6.20%)	-		
	c. Para 5 and above	101(78.21%)	-		
3	Booking status:				
	a. Booked	31(24.03%)	3.1612	69.59	0.001
	b. Unbooked	98(75.96%)			
4	Labour characteristics:				
	A: Mode of onset of labour:				
	I. Spontaneous	77(59.68%)	1.4807	9.6899	0.05
	ii. Induced	52(41.31%)			
	B: Status of membranes at admission:				
	I. Intact	21(16.27%)	0.1944	117.34	0.001
	ii. Absent	108(83.72%)			
	C: Mode of labour				
	I. vaginal	78(60.46%)	-	80.8371	0.001
	ii. Instrumental vaginal	22 (17.05%)			
	iii. Caesarean section	29(22.48%)			
	D: Place of delivery:				
	I. In hospital	34 (26.35%)	2.7941	57.6899	0.001
	ii. Referred from periphery	95 (73.64%)			

Highly affected population were> 31years of age group, para>5, unbooked cases, with spontaneous onset of labour, prolonged absent of membranes, spontaneous vaginal delivery

**Table-II:** Investigations (N= 129

*S. No*	*Investigations*	*No of Cases (%)*	*Relative Risk*	*Chi Square Test*	*P Value*
1	Blood complete picture:				
	A:Haemoglobin level:				
	I. up to 5 gram%	26(20.15%)		1.2978	0.5
	ii. between 6-8gram%	29(22.48%)			
	iii. between 8.5 to 9 grams %	74(57.36%)			
	B:Total leukocyte count:				
	i.7000-11000/cu.mm^2^	36 (27.90%)	2.5833	50.3720	0.001
	ii .>11000 cu.mm^2^	93 (72.09%)			
2	High vaginal swab culture and sensitivity:				
	Yes	72	1.2631	3.4883	0.05
	No	57			

(Majority of these women were with low haemoglobin level, and with raised total leukocyte count

**Table-III:** Clinical spectrum and maternal morbidity & mortality (N=129).

*S. No*	*Clinical Spectrum and Maternal Morbidity & Mortality*	*No of Cases*	*%*
1	Clinical Spectrum		
	I. Fever	117	90.69
	ii. Abdominal distention	28	21.70
	iii. Wound infection	51	39.53
	iv. Prolonged hospital stay (>10 days)	109	85.82
2	Maternal Morbidity & Mortality:		
	I. Disseminated intravascular coagulation	23	17.82
	ii. Septicemia	35	27.13
	iii. Death	11	8.52

Common symptoms were fever, wound infection; complications were septicemia and disseminated intravascular coagulation.)

## DISCUSSION

In this study the puerperal sepsis was highly reported in women of above 31 years of age group 84 (65.11%), and unbooked grand multiparous101 (78.29%). Worldwide in under developed countries this is the group of women who suffers a lot with infective morbidities, this can be due to poverty, illiteracy, malnutrition, they start their pregnancy in poor condition and having low resistance for the infection, these women do not usually seek ante natal checkup or contraceptive advise. In this study most of the cases were referral from outside 95 (73.64%), and had UN necessary labour induction 52 (41.3%) with improper sterilization by UN skilled personals this can be the reasons for their infective morbidities. Majority of these women 108 (83.72%) were having prolonged rupture of membranes at the time of admission, in these women second stage of labour was prolonged so the rate of second stage intervention by emergency Caesarean section 29 (22.48%) as well as by instrumental delivery was high 22 (17.05%), same is reported by Shamshad et al^11^ and other studies by Seale AC et al^12^ and Hussein J et al^13^ that specific interventions necessary for prevention and treatment of infection are good hand hygiene, use of antiseptic solution and appropriate antibiotic coverage. Increasing concerns of hospital and health care associated with infection control are currently recorded in many medical disciplines even in high income industrialized countries^14^ with this experience there is increasing trends of utilization of health facilities^15^ for prevention and control of infection, there is strong need of good implementation of established infection control programs at all health facilities in our country.

The infection control measures requires proper education, improvements of guidelines and various technologies and introduction of new clinical guidelines.^16^ Systemic review have not however found an established link between use of hand hygiene products and reduction in nosocomial infections.^17^ The application of antiseptic washes to the vaginal area during labour has received much current interest but there is insufficient evidence of its effectiveness in preventing maternal infection.^18^ In our part of world there is strong need of health education and continuous work in all aspects for improvement of maternal health.

In this study high morbidities seen were septicemia in 35 (27.13%) cases, disseminated intravascular coagulation in 23 (17.82%) cases and mortality rate was 11 (8.52%), same is reported by other national study.^19^ The high rate of morbidity and mortality could be late referral, and operative interventions in established infected malnourished women Characteristic problems related to infection control in developing countries include bad antibiotics prescribing practices, poorly functioning laboratory services, lack of surveillance data and suboptimal design or construction of buildings and water and sanitation systems. Overcrowding of facilities and insufficient number of health workers are commonly noted. Increased bed numbers, nurse to patient ratio and bed space are known to have negative effects on infection transmission. Managers role are not well specified, which contribute to the poorly quality of services.^20^^,^^21^ Prophylactic antibiotics during operation reduces endometritis by 66-75% and also reduces rate of wound infection.^22^ In our part of world considering all these factors proper education, training regarding anti septic techniques and proper antibiotic cover will improve a lot.

## CONCLUSION

The frequency of common preventable risk factors was high like low standard personal hygiene, obstetrics care, poverty, lack of knowledge of utilization of health care facilities available, unplanned pregnancies, unnecessary induction and delivery by un skilled personals. This all results in severe life threatening complications such as septicaemia disseminated intravascular coagulation as well as maternal death.
